# Persistent hyperammonia and altered concentrations of urea cycle metabolites in a 5-day swine experiment of sepsis

**DOI:** 10.1038/s41598-021-97855-7

**Published:** 2021-09-16

**Authors:** Manuela Ferrario, Roberta Pastorelli, Laura Brunelli, Shengchen Liu, Pedro Paulo Zanella do Amaral Campos, Daniela Casoni, Werner J. Z’Graggen, Stephan M. Jakob

**Affiliations:** 1 Department of Electronics, Information and Bioengineering, Politecnico di Milano, Milan, Italy; 2 Istituto di Ricerche Farmacologiche Mario Negri IRCCS, Milan, Italy; 3 Department of Intensive Care Medicine, Bern University Hospital, University of Bern, Bern, Switzerland; 4 Department of Intensive Care Unit, Hospital Israelita Albert Einstein, São Paulo, Brazil; 5 Department for Bio Medical Research, Experimental Surgery Facility, University of Bern, Bern, Switzerland; 6 Departments of Neurology and Neurosurgery, Bern University Hospital, University of Bern, Bern, Switzerland

**Keywords:** Metabolomics, Biomarkers, Experimental models of disease

## Abstract

We measured plasma and cerebrospinal fluid (CSF) metabolite concentrations in a 5-day porcine sepsis model of fecal peritonitis. The objectives were: (i) to verify whether the expected pathways that had emerged in previous studies pertain only to the early inflammatory response or persist for the subsequent days; (ii) to identify metabolic derangements that arise later; (iii) to verify whether CSF metabolite concentrations were altered and if these alterations were similar to those in the blood or delayed. We observed an early response to inflammation and cytokine storms with alterations in lipid and glucose metabolism. The arginine/asymmetric dimethylarginine (ADMA) and phenylalanine/tyrosine balances changed 24 h after resuscitation in plasma, and later in CSF. There was a rise in ammonia concentration, with altered concentrations of metabolites in the urea cycle. Whether persistent derangement of these pathways have a role not only on short-term outcomes but also on longer-term comorbidities, such as septic encephalopathy, should be addressed in further studies.

## Introduction

Sepsis is a life-threatening disease caused by a dysregulated host response to infection. This condition can deteriorate to septic shock in which circulatory and cellular/metabolic abnormalities may substantially increase the mortality risk^1,2^. In survivors, organ dysfunction, including dysfunction of the brain, may have long-term consequences on physical and mental health, and on quality of live^3,4^.

The uncontrolled inflammatory response in sepsis and septic shock condition activates or alters several metabolic pathways related to complex biological communication between vital organs. However, the temporal evolution of sepsis is variable, and the phenotype dependent, among others, on source of sepsis, amount fluid losses and elapsed time until diagnosis. Accordingly, time course and magnitude of metabolic abnormalities in response to the septic insult are difficult to interpret in retrospect.

The aim of the present study was to measure the metabolic derangements induced by sepsis by quantitative metabolomics analysis of plasma and cerebrospinal fluid (CSF) in a clinically relevant 5-day animal model. This allows assessment of metabolites’ concentrations at health, before sepsis occurs, and then repeatedly at exactly defined time points after the septic insult and from different compartments. With this information, precise determination and quantification of early and late metabolic pathways in sepsis is possible, and also whether the pathways are similar, different or just delayed in cerebrospinal fluid compared to blood. Future studies may then investigate, whether certain pathways can beneficially be modulated or specific compounds used as biomarkers for sepsis and or brain dysfunction evolution.

## Results

### Clinical data

**Figure 1:**
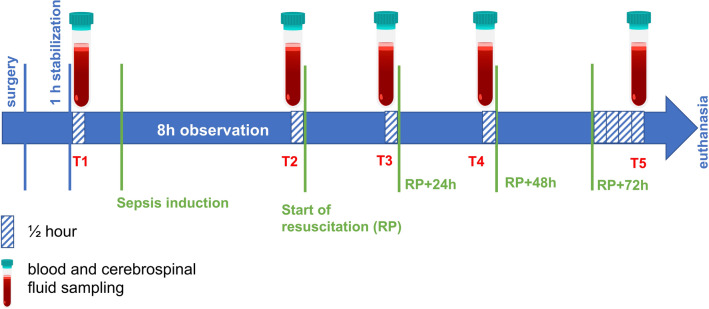
Diagram of study protocol. After installation of flow probes and catheters, all animals were allowed 1 h of stabilization. Within 30 min after stabilization blood and CSF were collected (T1) and this was considered the baseline condition. Sepsis was induced by autologous fecal solution followed by 8 h of observation without treatment. Then resuscitation started and was maintained for approximately 76 h. Blood and CSF samples were collected approximately 30 min before resuscitation (T2) when sepsis had developed, and approximately 24, 48 and 74 h (T3, T4 and T5) after resuscitation started, for a total of five time points.

Twenty anesthetized, mechanically ventilated pigs were randomized to fecal peritonitis or sham (n = 10/group): Blood and cerebrospinal fluid (CSF) samples were collect at baseline, 8 h of observation after the insult without sepsis treatment, and sequentially within 24, 48 and 74 h after resuscitation, for a total of five time points. The protocol is illustrated in Fig. 1. The experimental model is consolidated and known to produce systemic inflammation and organ failure^5^.

All animals survived the experiment. The clinical data are reported in the Supplemental Material (Fig. S1, Table S3) for readers’ convenience as they were already published^5^. Briefly, after induction of fecal peritonitis, before resuscitation animals had low cardiac output and blood pressure. Hemoconcentration and low filling pressures suggested capillary leakage with fluid sequestration. Resuscitation then produced the typical hyperdynamic state with increased temperature, heart rate and cardiac output (CO). Septic animals received more Ringer’s lactate, norepinephrine and glucose to maintain hemodynamic stability and homeostasis, but at the same time, had lower urine output and higher positive fluid balance than sham pigs^5^. At T2/T3, platelets and leucocytes concentration were lower in septic than in sham animals, but band neutrophils percentage remained significantly higher in the septic group until the end of the experiment, although decreasing from T2 to T5 (Fig. S2, Table S3).

### Plasma metabolomics landscape

We employed mass spectrometry-based quantitative metabolomic profiling to unambiguously identify and quantify lipids, amino acids, biogenic amines and acylcarnitines in the plasma of animal groups. In total, 124 metabolites were quantified out of the measurable 186 (see Table S2).

### Multivariate analyses of plasma metabolomics data

**Figure 2:**
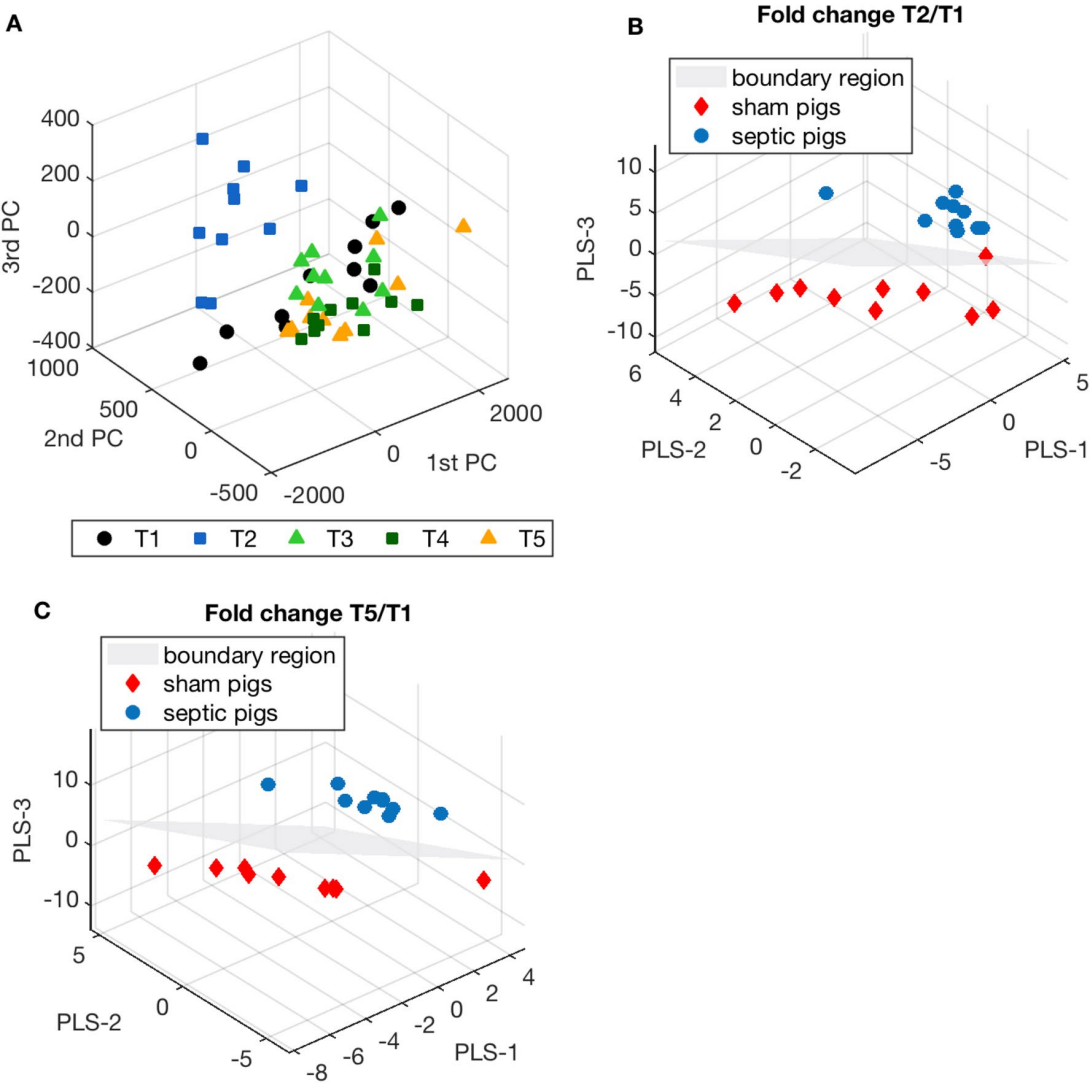
(**A**) MultiLevel simultaneous component analysis (MLSCA) on I = 10 septic pigs, considering all the plasma metabolite concentrations (J = 124) and all the time points: baseline before sepsis induction (T1), sepsis before resuscitation (T2) and 24, 48 and 74 h after resuscitation (T3, T4 and T5). (**B**) Partial Least Square Discriminant Analysis (PLS-DA) on the first 40 ranked features filtered by mRMR technique, computing the fold-change T2/T1 of the identified metabolites and taking the septic group as the target class for classification. (**C**) Same analysis repeated for the first 40 ranked features filtered by mRMR technique, obtained by computing the fold change T5/T1 of the identified metabolites.

Figure 2A shows the MultiLevel Simultaneous Component Analysis (MLSCA). In the multilevel models the differences between subjects and within subjects are separated. We ran a MLSCA model on 10 septic animals (I = 10) considering five time points (K = 5) and all the quantified metabolites (J = 124). There was a clear metabolic derangement after sepsis (at T2) with the associated symbols well separated and the symbols for the other time points overlapped.

Successively, after filtering the features in order to reduce redundancies and collinearities, we ran partial least square discriminant analysis (PLS-DA) taking into consideration the T2/T1 and T5/T1 fold change to characterize the main differences in the metabolic pathways between septic and sham animals after the insult and at the end of the experiment. Figure 2B,C illustrate the PLS-DA. The models correctly separate the two groups of animals. For each model, we computed the VIP scores to rank the variables according to their importance in the classification (Table 1).

**Table 1:** VIP scores of the PLS-DA model based on the fold-changes between T2 and T1 and between T5 and T1 plasma metabolite concentrations.

Fold-change T2/T1		Fold-change T5/T1	
Features	VIP	Features	VIP
Putrescine	1.60	ADMA	1.519
Gln	1.43	total DMA	1.494
Ala	1.30	Asp	1.458
lysoPC a C17:0	1.18	Asn	1.401
PC aa C38:4	1.13	Thr	1.394
PC aa C36:4	1.11	alpha-AAA	1.259
PC aa C36:1	1.10	Trp	1.130
PC aa C36:3	1.10	Putrescine	1.115
PC aa C38:6	1.09	C18:1	1.090
PC ae C38:0	1.09	Met	1.051
lysoPC a C18:1	1.07	PC ae C40:1	1.045
Cit	1.07	PC ae C38:0	1.032
Kynurenine	1.07	PC aa C36:5	1.026
lysoPC a C18:0	1.07	PC aa C38:6	1.001
lysoPC a C20:4	1.06	Orn	0.997
Phe	1.05	Arg	0.955
PC aa C34:2	1.05	PC aa C38:4	0.951
PC ae C40:1	1.05	C0	0.949
PC aa C40:4	1.04	lysoPC a C18:0	0.946
lysoPC a C18:2	1.03	t4-OH-Pro	0.926
PC ae C36:2	1.03	lysoPC a C16:0	0.917
lysoPC a C16:1	1.02	Leu	0.915
PC ae C38:4	1.01	lysoPC a C18:1	0.914
PC ae C36:3	1.00	Tyr	0.910
PC ae C42:2	0.97	Val	0.908
PC ae C38:3	0.94	PC ae C36:5	0.905
PC aa C38:3	0.94	PC ae C32:2	0.900
PC ae C40:6	0.93	SM C16:1	0.864
PC aa C36:5	0.92	lysoPC a C17:0	0.858
PC ae C38:6	0.92	PC ae C42:2	0.833
PC aa C40:3	0.91	Serotonin	0.823
PC aa C32:1	0.88	PC ae C40:4	0.784
PC ae C32:2	0.84	PC ae C40:5	0.774
PC aa C30:2	0.80	PC ae C40:6	0.761
PC aa C42:1	0.67	His	0.748
PC ae C40:4	0.66	PC aa C38:5	0.735
PC aa C38:5	0.60	PC aa C40:6	0.722
PC aa C40:6	0.39	PC aa C38:1	0.710
Spermidine	0.33	SM C18:0	0.709
SM C22:3	0.27	PC ae C44:6	0.535

The models were built on the first 40 ranked features selected by a filter technique. A feature with a variable importance (VIP) score higher than 1 is considered significant in the classification model.

**Figure 3:**
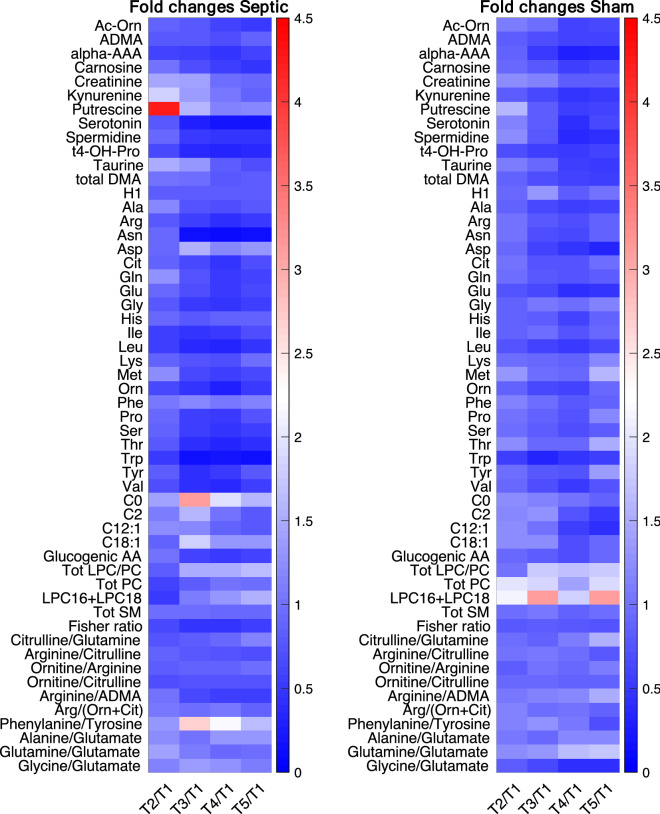
Heat map for the mean values of fold changes in concentrations of plasma metabolites at all the experimental time points and baseline (T1) in septic and sham animals separately. Lipids are summed up and ratios associated to the pathways of urea and ammonia detoxification, arginine and phenylamine. For the acronyms see Table S1; *AA* amino acids, *Glucogenic AA* sum of all glucogenic amino acids (Alanine Arginine, Asparagine, Aspartic acid, Cysteine, Glutamic acid, Glutamine, Glycine, Histidine, Methionine, Proline, Serine, Valine), *Tot LPC/PC* ratio of the total amount of lysoPC and PC species, *Tot PC* total amount of PC species, *LPC16 + LPC18* total amount of the most abundant lysoPC (lyso PC 16:X and lyso PC 18:X), *Tot SM* total amount of sphingomyelin species, *Fisher ratio* ratio of branched-chain AAs (leucine, valine, isoleucine) to aromatic AAs (phenylalanine, tyrosine).

These preliminary analyses indicated that lysophosphatidylcholines (LPC), phosphatidylcholines (PC) and the ratio between the amounts of LPCs and PC were reduced after the development of sepsis and did not recover after resuscitation (Fig. 3, Supplementary Fig. S3). The heat map of the metabolite concentration trajectories highlighted putrescine, an intermediate of polyamine synthesis, with a marked increase at T2 in the septic animals, then recovering quite fast (Fig. 3); it was at the first rank of the multivariate model.

We then focused on specific pathways characterizing the acute phase of shock and the phases of resuscitation, and clearly emerged from the multivariate analyses.

### Energy-related metabolic pathways and liver function

The inflammatory insult induced in septic animals produced, as expected, a significant increase in lactate in different circulatory districts from T2 (Table S3, Fig. S4), remaining significantly higher than in sham animals until the end of the experiment, although the values are less than 2 mmol/L.

We then focused on specific metabolic species related to glucogenesis as the energy imbalance in sepsis is crucial from the early phases. Glucogenic amino acids (alanine, arginine, asparagine, aspartic acid, cysteine, glutamate, glutamine, glycine, histidine, methionine, proline, serine, valine) can give a picture of glucose production and its related pathways.

Glutamine and alanine, two glucogenic metabolites, appeared in the first ranks of the VIP score of the model on T2/T1 fold-changes (Table 1).

**Figure 4:**
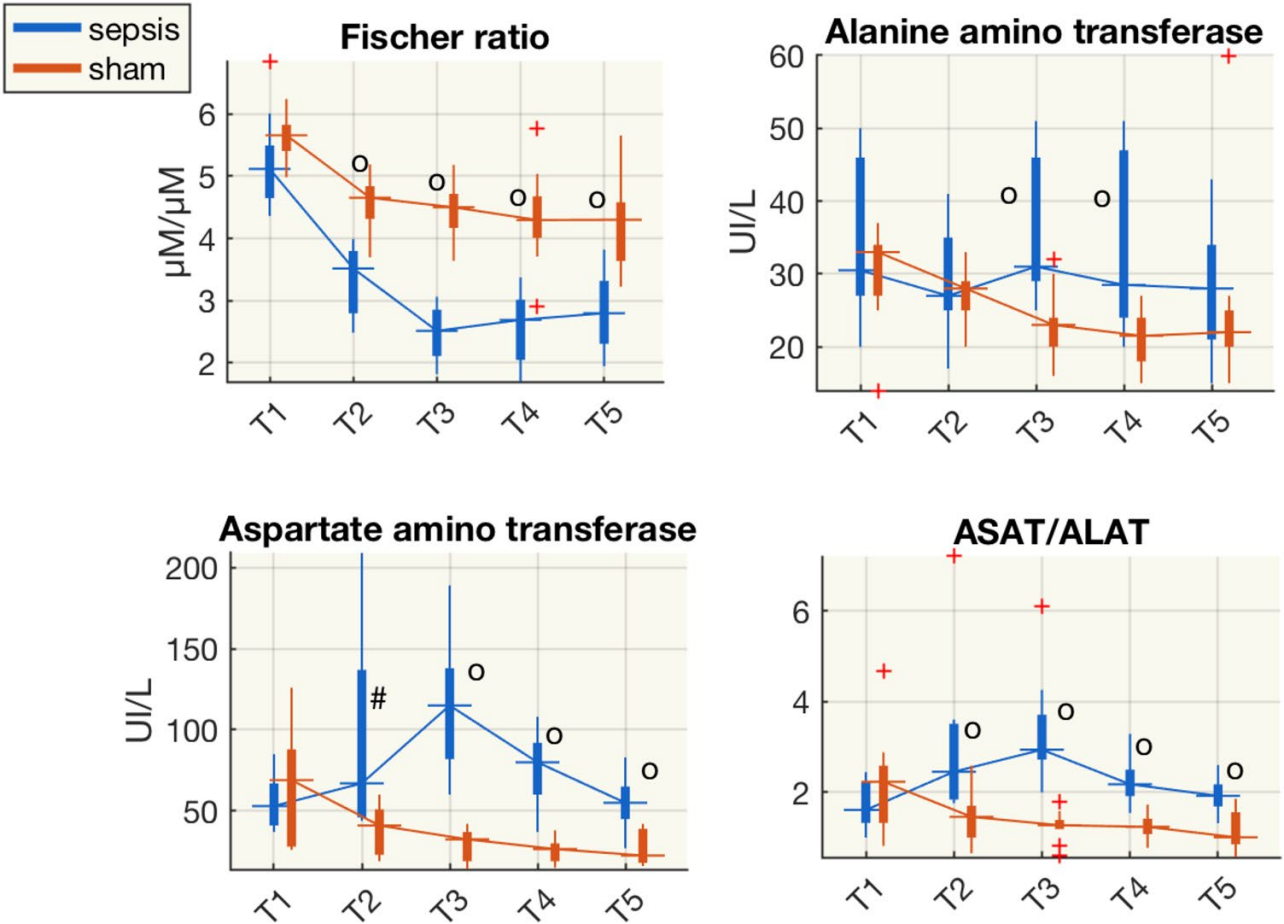
Boxplot of the Fischer ratio (ratio of concentrations of branched-chain AAs-leucine, valine, isoleucine—to aromatic AAs-phenylalanine, tyrosine), concentrations of alanine amino transaminase (ALAT), aspartate amino transaminase (ASAT), and the ASAT/ALAT ratio from plasma samples at each time point for the sham and septic pigs. ^#^p < 0.05, ^o^p < 0.01(Wilcoxon rank-sum test) sham vs shock; *µM,* µmol/L.

We also computed the Fischer ratio, which is the molar ratio of branched-chain AAs (BCAAs: leucine, valine, isoleucine) to aromatic AAs (phenylalanine, tyrosine), often used as an indicator of liver damage^6^. The Fischer ratio was approximately halved in septic pigs and was significantly lower than in sham animals from T2 (Fig. 4). At the same time, there was an increase in transaminases, significantly higher in septic animals. Aspartate transaminase (ASAT) increased more than alanine transaminase (ALAT), as shown by the ASAT/ALAT ratios (Fig. 4). Among the standard indices of liver functionality bilirubin values were significantly higher in septic than in sham animals only at T5 (Table S3). These biomolecules trajectories hinted at liver dysfunction, which could have started with the insult, i.e. several hours earlier, as the patterns of the metabolites and transaminases suggest.

### Urea cycle and ammonia detoxification pathways

The urea cycle converts ammonia to urea and is essential for the ammonia detoxification. In the multivariate analyses citrulline, glutamine, aspartate and asparagine were at the first ranks of the VIP scores for the models based on metabolite fold-changes (Table 1), and they are key metabolites in ammonia pathways. Therefore, we analyzed the urea cycle metabolites, and computed the molar ratios that indicate a measure of the transformation reactions. In the hepatic cytosol, citrulline reacts with aspartate to form argininosuccinate. Argininosuccinate lyase (AL) cleaves argininosuccinate to form fumarate, which is oxidized in the tricarboxylic acid cycle (TCA), and arginine, which is then hydrolyzed to urea and ornithine via hepatic arginase (ARG). In addition, ammonia forms glutamine via glutamate. Glutamine synthetase (GS) is an essential enzyme in the metabolism of nitrogen, catalyzing the condensation of glutamate and ammonia to form glutamine. Similarly, asparagine synthetase (ASNS, or aspartate-ammonia ligase) is a cytoplasmic enzyme that generates asparagine from aspartate. Glycine too can be an active ammonia-genesis amino acid leading to changes in plasma and brain ammonia levels.

This complex urea biochemical network led us to investigate arginine, asparagine, aspartate, citrulline, ornithine, glutamate, glutamine, glycine and the following ratios: citrulline/glutamine, arginine/citrulline, ornithine/arginine, ornithine/citrulline, arginine/(ornithine + citrulline), as measures of arginine bioavailability, glutamine/glutamate, glycine/glutamate. This information was paralleled with the ammonia values and the ratio of ammonia to urea cycle related metabolites.

**Figure 5:**
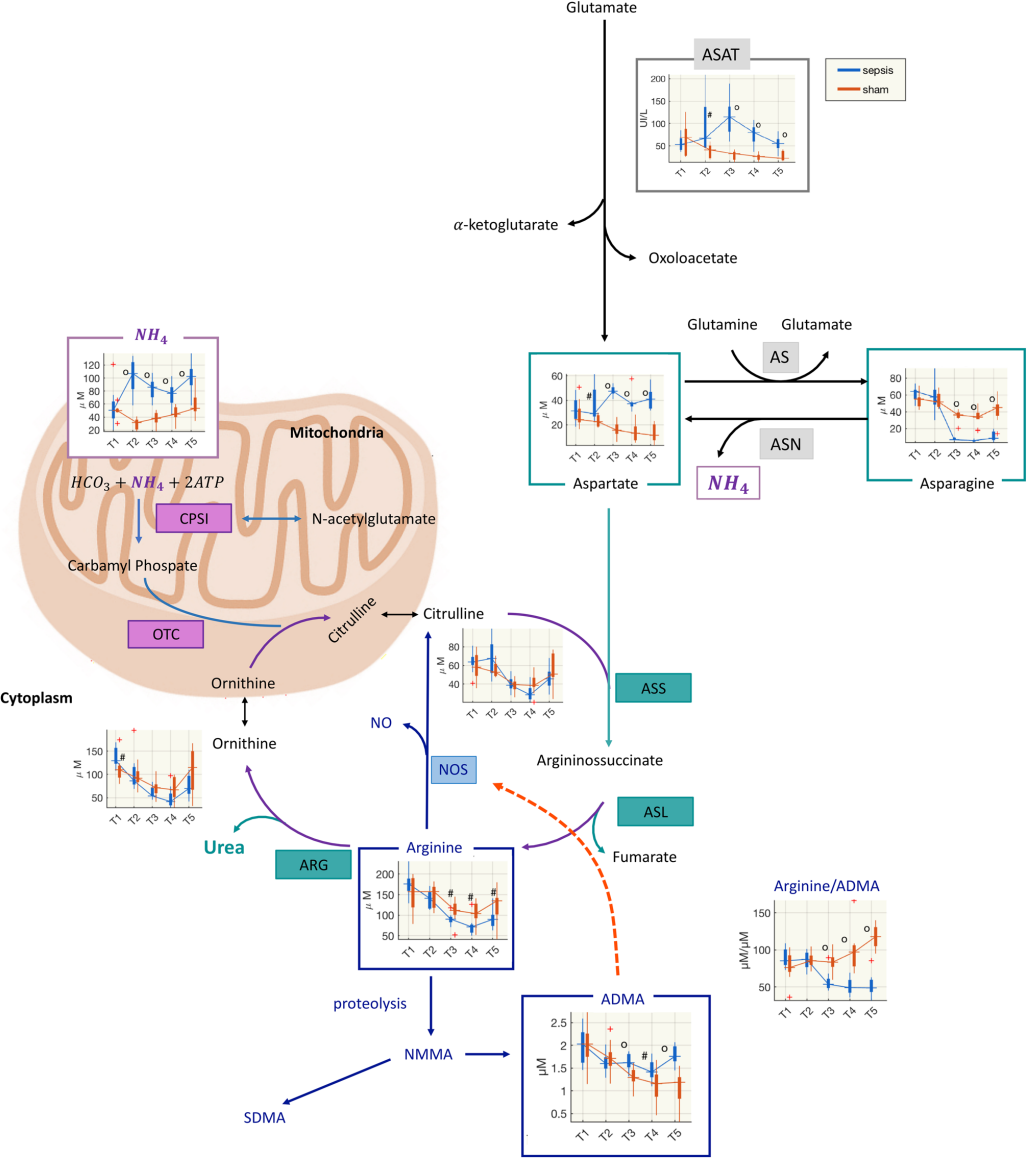
Diagram of urea cycle, NOS and degradation of arginine and asparagine. The urea cycle converts ammonia to urea. The cycle “begins” in hepatic mitochondria, where ammonia (NH_4_), HCO_3_ and ATP form carbamyl phosphate in a reaction catalyzed by carbamyl phosphate synthetase (CPSI). *N*-acetylglutamate (NAG), formed from glutamate and acetyl-CoA via *N*-acetylglutamate synthetase is an obligatory effector of CPSI and an important regulator of ureagenesis. Various influences, including dietary protein, arginine and corticosteroids, augment the concentration of NAG. Carbamyl phosphate condenses with ornithine to yield citrulline in the ornithine transcarbamylase (OTC) reaction. In the hepatic cytosol, citrulline reacts with aspartate to form argininosuccinate, catalyzed by argininosuccinate synthetase (ASS). Argininosuccinate lyase (ASL) cleaves argininosuccinate to form fumarate, which is oxidized in the tricarboxylic acid cycle (TCA), and arginine, which is hydrolyzed to urea and ornithine via hepatic arginase (ARG). Arginine is also the precursor for the biosynthesis of nitric oxide (NO). NO is synthesized via nitric oxide synthase (NOS), this pathway draws arginine from the urea cycle, alternatively, arginine may undergo proteolysis to become asymmetric dimethylarginine (ADMA), which acts as an endogenous competitive inhibitor of NOS and causes local vasoconstriction. ADMA has been proposed as an independent biomarker of endothelial dysfunction: the arginine/ADMA ratio provides information on arginine bioavailability for production of NO^35^. Symmetric dimethylarginine (SDMA) is another methylated analogue of arginine found in humans. Aspartate transaminase (ASAT) catalyzes the reversible transfer of an α-amino group between aspartate and glutamate. The enzyme asparagine synthetase (AS) produces asparagine and glutamate from aspartate and glutamine. In the asparagine synthetase reaction, ATP is used to activate aspartate. On the other hand, the enzyme asparaginase (ASN) catalyzes the hydrolysis of l-asparagine into aspartate and ammonia. *NMMA, N*-methylarginine.

Ammonia was significantly higher from the end of the observation period after the insult (T2) until the end of the experiment. At T2 ammonia in septic pigs was more than three times that in of sham group (Fig. 5, Table S3, Supplementary Fig. S5).

Sham animal showed an early increase in glutamine at T2, then glycine was significantly lower from T3, and the glutamine/glutamate ratio dropped at T4 and T5. This suggests that the reactions of the glycine cleavage system shifted in the direction of ammonia production from glycine. The conversion of glutamate and ammonia into glutamine also seemed involved, as the ratio of glutamine to ammonia was significantly lower in septic animals at T2, T3 and T4.

Compared to sham animals the concentration of aspartate was significantly higher while the concentration of arginine and the arginine/citrulline ratio were significantly lower after the insult. The bioavailability of arginine did not significantly differ in the two groups and was quite stable. Asparagine was significantly lower from T3. These results hint at less conversion of aspartate to produce asparagine, by utilizing ammonia (Fig. 5).

### Nitric oxide-related pathways: asymmetric dimethylarginine (ADMA) and phenylalanine

Multivariate analyses indicated that asymmetric dimethylarginine (ADMA) and phenylalanine were in the top of the VIP score for the models based on metabolites changes (Table 1).

**Figure 6:**
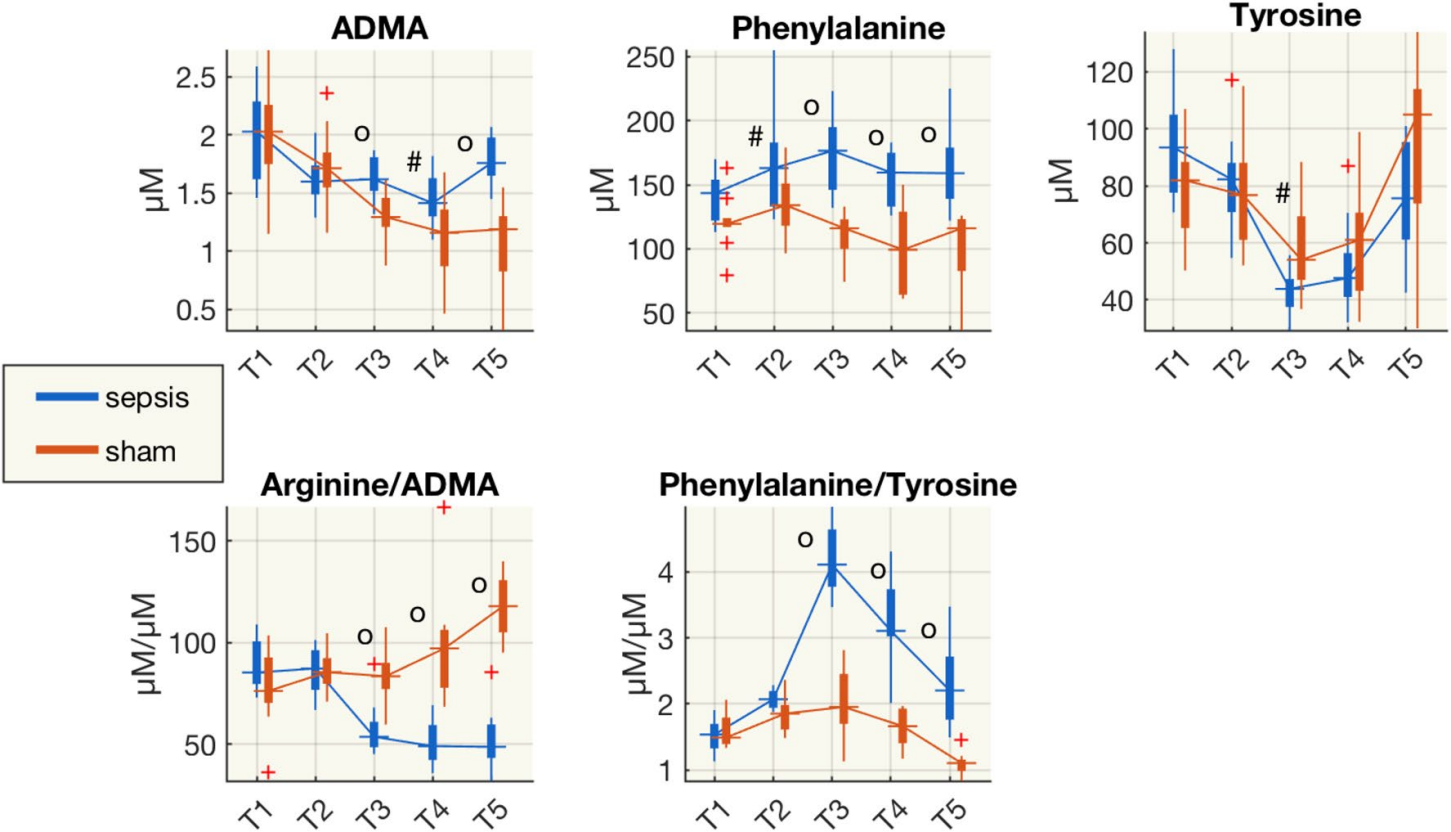
Boxplot of the concentrations of asymmetric dimethylarginine (ADMA), phenylalanine, tyrosine, arginine/ADMA and phenylalanine/tyrosine from plasma samples at each time point for sham and septic pigs. ^#^p < 0.05, ^o^p < 0.01 (Wilcoxon rank-sum test) sham vs shock; *µM,* µmol/L.

In our experiment, the ratio of arginine to ADMA decreased in septic animals and was significantly lower than in sham group from T3 (Fig. 6). This might be explained by a more pronounced decrease of the arginine concentration (Fig. 5, Supplementary Fig. S5) than ADMA, which was significantly higher than in the sham group and stable during the experiment.

Phenylalanine is a precursor for tyrosine, the monoamine neurotransmitters dopamine, which is the metabolic precursor of norepinephrine (noradrenaline) and epinephrine (adrenaline). The phenylalanine/tyrosine ratio was significantly higher in septic animals from T3 and this might be due to a decrease in tyrosine and an increase in phenylalanine, as these values were significantly higher in the septic group than sham animals.

### Cerebrospinal fluid metabolomics landscape

In 14 of the 20 pigs (6 sham and 8 septic pigs) cerebrospinal fluid (CSF) was collected and analyzed using the same technological platform as for plasma metabolomics. Fifty-four (54) metabolites were quantified, out of 186 measurable.

**Figure 7:**
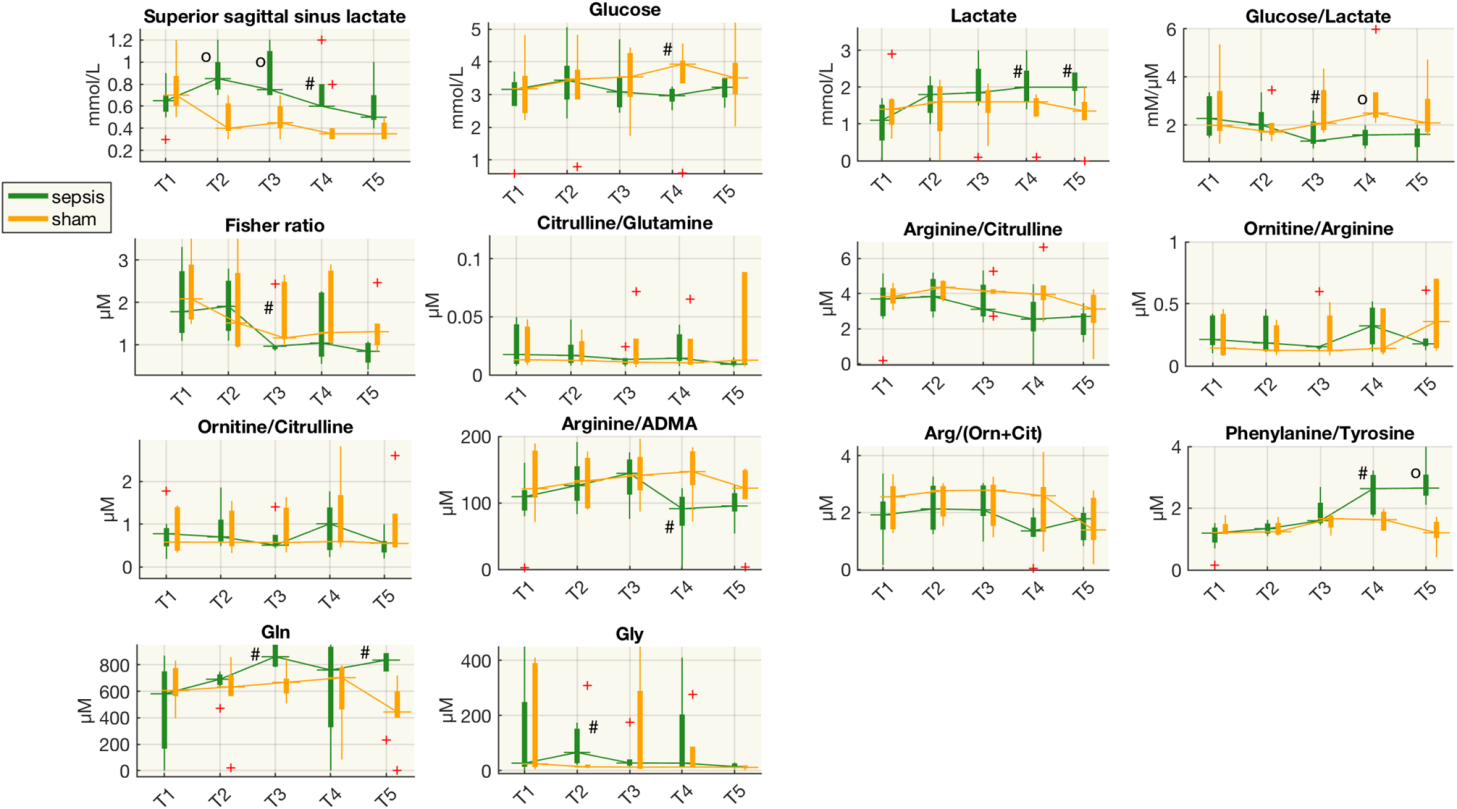
Boxplot of concentrations of several species from CSF samples at each time point for the sham and septic pigs. ^#^p < 0.05, ^o^p < 0.01 (Wilcoxon rank-sum test) sham vs shock; *µM,* µmol/L; *mM,* mmol/L; *Gln,* glutamine; *Gly,* glycine.

The metabolite concentrations in CSF varied widely, but with few significant differences between the two groups. However, there were similar trends for some of the biochemical pathways previously highlighted in plasma metabolome. For example, the phenylalanine/tyrosine ratio in the CSF increased and was significantly higher than in sham animals from T4 (Fig. 7), while in plasma it was higher from T3. Similarly, the arginine/ADMA ratio in CSF was significantly lower in septic animals at T4, but from T3 in plasma. Finally, there was CSF glutamine higher in septic animals at T3 and T5 than in shams.

Lactate in CSF was significantly higher at T4 and T5, later than in the arterial vessels. However, lactate in the superior sagittal sinus (SSS) was significantly higher in septic animals at T2, T3 and T4 and decreased at T5, to levels similar to those in sham animals. The balance between glucose and lactate was altered only slightly as glucose was significantly higher at T4 and the glucose/lactate ratio was significantly lower at T3 and T4.

## Discussion

The main feature of this experiment is the long observation time after sepsis that enabled us to determine magnitude and time course of a large number of metabolic changes in responses to the inflammatory insult and the subsequent standardized sepsis treatment.

After induction of fecal peritonitis, before resuscitation, the animals had low cardiac output and blood pressure. Hemoconcentration and low filling pressures suggested capillary leakage with fluid sequestration. Resuscitation then produced the typical hyperdynamic state with increases in temperature, heart rate and cardiac output (CO) (Figs. S1, S2). The leukocyte concentration (WBC) at T2 was lower in septic than in sham animals and band neutrophils remained significantly higher in the septic group up to the end of the experiment, indicating that the inflammatory state was not fully resolved.

In comparison to similarly instrumented and anesthetized control animals, we observed in septic animals early metabolic changes, which rapidly resolved (sugars, alanine, glucogenic amino acids), partly resolved (lactate, arginine, lysophosphatidylcholines), or were persistent (phenylalanine, aspartate, asparagine, ADMA).

As expected and previously reported^7,8^, sepsis altered the overall lipidome (Fig. 3, Supplementary Fig. S3). Our results confirmed that the lysophosphatidylcholines (LPCs) and PCs were reduced just after the development of sepsis and did not or only partly recover after resuscitation. This is in accordance with their complex role in the first inflammatory response: LPCs enhance the suppressive function of CD4C, CD25C regulatory T cells (Tregs) in anti-inflammatory signaling^9^. LPCs have been identified as prognostic biomarkers of mortality or non-responsiveness to therapy^10^. Plasma LPCs levels have been low in sepsis patients and systemic treatment with LPCs has proved therapeutic in rodent models of sepsis^11^.

We corroborated previous findings from our group and others^12–15^ on hepatic dysfunction in the early phase of sepsis. It comes as no surprise that liver transaminases ASAT and ALAT rose significantly within 48 h after resuscitation started. Although the transaminases partially decline during resuscitation, they never recovered to the levels in the sham group (Fig. 4), while bilirubin only increased later during the disease. Liver dysfunction was also supported by the Fischer ratio^6^. The ratio of the concentrations of branched chain amino acids (BCAAs) to aromatic amino acids (AAAs)—often decreased in patients with hepatic failure—dropped 24 h after resuscitation in the sepsis group and never rose in 74 h after resuscitation, suggesting that subclinical hepatic alterations^6,16^ probably started with the septic insult, and did not recover in the post-resuscitation time. The higher plasma alanine and glucogenesis AAs (Fig. S4) and the increase of lactate concentration in septic animals in several circulatory districts, is compatible with an imbalance of gluconeogenesis-glycolysis in the liver, and, at later time points, also in the brain (Fig. 7).

The liver dysfunction was further highlighted by the time-course of the increase of circulating ammonia and decrease in intermediates of the urea cycle, involving enzymes that together are only found in the liver (Fig. 5). We speculate that, as a first response to the inflammatory insult, ornithine might have been catalyzed by ornithine decarboxylase to synthesize putrescine, which was in fact substantially increased in the septic group (Fig. 3). Putrescine has an important role in the synthesis of polyamines that exert pleiotropic biological activities, including modulation of cell signaling, cell growth and apoptosis^17^. The marked increase in ammonia in septic animals started immediately after sepsis development and did not recover during the 74 h after resuscitation observation period (Fig. 5, Supplementary Fig. S5). Serum ammonia levels were an independent risk factor for the prognosis of septic patients in a recent study^18^. The urea cycle converts ammonia to urea and the starting point of the cycle is in the hepatic mitochondria. It seems plausible that a decrease in the ability to detoxify ammonia in the liver increases ammonia concentrations in the brain. In organotypic slice models, ammonia increased glutamine concentrations and caused inflammation and swelling of brain cells^19^. Our results showed higher levels of glutamine in CSF samples of septic animals, which may corroborate the hypothesis related to ammonia’s neurotoxic effect.

In this study we did not consider the ATP/ADP/AMP axis and tricarboxylic acid cycle (TCA) cycle intermediates, such as succinate, fumarate and citrate, which play a crucial role in the response to septic insults^20^. Pyruvate dehydrogenase complex (PDC) is a master metabolic regulator controlling the conversion of pyruvate to acetyl-CoA in the mitochondria and its inactivation contributes to the metabolic reprogramming that occurs in immune cells in response to inflammation^21,22^.

Pyruvate dehydrogenase kinase (PDK) phosphorylates PDC and inhibits the conversion of pyruvate to acetyl-CoA. The decreased synthesis of acetyl-CoA limits TCA cycle-supported anabolism and converts the high energy demands to a tolerable, catabolic low-energy supply state^23^. The loss of anabolic biosynthetic pathways develops within hours of the onset of inflammatory shock and TCA cycle shifts from intermediate cis-aconitate to itaconate. Itaconate directly inhibits succinate dehydrogenase (SDH), leading to disruption of oxidative phosphorylation and decreased ATP synthesis^24^. If this negative feedback loop of repressed anabolism persists, a life-threatening clinical syndrome of immunometabolic paralysis develops.

Modulation of TCA cycle seems to affect urea cycle intermediates as well. In a monocyte culture model of severe acute inflammation that simulates sepsis reprogramming, the inhibition of PDK increased urea cycle activity, with elevated arginine, ornithine, citrulline, aspartate and possible repletion of the TCA cycle intermediate fumarate, while decreasing itaconate, and increasing anaplerotic catabolism of branched-chain amino acids (BCAAs)^21^.

Finally, our septic animals had a persistent increase of plasma asymmetric dimethylarginine (ADMA) and a concomitant reduction of the arginine/ADMA ratio, starting from T3. ADMA is an endogenous inhibitor of eNOS and the arginine/ADMA ratio is considered an indicator of NO bioavailability^25^. A low arginine/ADMA ratio can indicate reduced NO production due to reduced availability of L-arginine to NOS^26,27^. We observed also a decreasing of arginine/ADMA ratio in the CSF of sepsis animals later time after resuscitation (T4), compared to plasma (T3) (Figs. 6, 7).

Furthermore, our results point to the dysregulation of the phenylalanine-tyrosine ratio (Phe-Tyr). This was a late response after the insult, and was also observed in the CSF—at an even later time point (Figs. 6, 7). Arterial concentrations of phenylalanine were also increased after intravenous lipopolysaccharide (LPS) infusion in healthy volunteers^28^. The liver is the main site of phenylalanine metabolism; therefore, deterioration of liver function may reduce this metabolism, raising systemic phenylalanine levels. The biosynthesis of Phe to Tyr mediated by phenylalanine hydrolase and the transformation of Tyr to Dopamine (DOPA) by tyrosine hydroxylase requires the tetrahydrobiopterin (BH4) as cofactor. Inflammation and immune activation affect BH4 availability, and as a consequence, the biosynthesis of phenylalanine and tyrosine is altered^29^. Moreover, BH4 modulates specific metabolic changes in inflammatory macrophages by the regulation of NO production. These include regulating respiratory function by changes in levels of the important TCA cycle metabolites, and inflammatory mediators, citrate, succinate, and itaconate^30^.

The impaired conversion of phenylalanine to tyrosine in the liver and the concomitant decrease in tyrosine affect the amino acids transport into the brain and its further conversion to its downstream metabolites dopamine, epinephrine and norepinephrine^31^. Currently the precise mechanisms responsible for the neurological effects of phenylalanine accumulation are unclear.

Finally, the interplay among different metabolic intermediates and cofactors as well as organ crosstalk, such as liver-brain axis, need further investigations.

## Conclusion

This study illustrates the timing of different pathways associated with the development of sepsis and resuscitation. Our results confirmed the early metabolic responses to inflammation and cytokine storms with alteration in lipid and glucose metabolism. Other pathways, which may have an impact on brain function, such as the phenylalanine/tyrosine balance, occurred after T3 in plasma and later in CSF. In addition, besides the increase in ammonia concentration, our results suggest an alteration in the urea cycle.

## Methods

### Animal experiment

The animal study was conducted according to the EU Directive 2010/63/EU for animal experiments and the ARRIVE guidelines for animal research, and was approved by the Animal Care Committee of the Canton of Bern, Switzerland (BE103/16). Details of the animals, anesthesia, instrumentation and experimental protocol were previously reported^5^.

Briefly, 20 mechanically ventilated, unconscious domestic pigs (weight: 39.8 ± 2.7 kg of both sex) were used. The pigs were sedated with ketamine (20 mg/kg) and xylazine (2 mg/kg) intramuscularly. General anesthesia was induced with midazolam (0.5–1.0 mg/kg) and atropine (0.02 mg/kg) intravenously, and maintained with propofol (4–10 mg/kg/h) and fentanyl (3–20 μg/kg/h). The depth of anesthesia was evaluated continuously with 1 derivation EEG, and observation of spontaneous movements, while nociception was checked hourly through toe pinching. After tracheal intubation, the pigs were mechanically ventilated in volume-controlled mode, initially with 30%, FiO_2,_ 5 cmH_2_O PEEP, 8 ml/kg tidal volume and 20 breaths/min respiratory rate. This was later adjusted with the goal to keep pH 7.35–7.45 and PaCO_2_ 35–45 mmHg.

With the pigs in sternal recumbency, a spinal catheter was percutaneously inserted to collect cerebral spinal fluid. Then, with the pigs in dorsal recumbency, a midline neck incision was made to allow placement of an arterial catheter (5F, Cordis AVANTI, Fremont, CA, USA) in the left carotid artery, a pulmonary artery catheter (8F, Edwards Lifesciences, Irvine, USA) via left external jugular vein, a venous catheter in the left internal jugular vein (5F, Cordis AVANTI, Fremont, CA, USA) for volume infusion, a triple-lumen catheter (7F, Arrow international, Inc, PA, USA) in the right internal jugular vein, and a flow probe (Transonic Systems Inc., Ithaca, NY, USA) around the right carotid artery to measure blood pressures, O_2_ saturation and flow. The pig was then turned into a left lateral position and craniotomy was performed to place a single-lumen oximetry catheter (4F, Edwards Lifesciences, Irvine, USA) in the superior sagittal sinus together with a pressure probe (Probe 3PS, Spiegelberg GmbH & Co. KG, Hamburg, Germany) to measure sagittal sinus and intracranial pressures, used for a separate study^5^. After midline laparotomy in dorsal recumbency, a catheter was inserted into the urinary bladder for drainage and measurement of urine output. Flows in the portal vein, hepatic artery and right kidney artery were measured by means of probes (Transonic Systems Inc., Ithaca, NY, USA) positioned around the vessels.

A single-lumen catheter was positioned in the portal vein through a mesenteric vein to measure portal vein pressure (5F, Cordis AVANTI, Fremont, CA, USA). An 8.5 F introducer was placed in the hepatic vein for a pulmonary artery catheter (8F, Edwards Lifesciences, Irvine, USA) to measure hepatic vein O2 saturation (ShvO_2_) and hepatic venous pressure (Phv). Another pulmonary artery catheter was placed in the right renal vein through the right femoral vein to measure renal vein O2 saturation and pressure. Before abdominal closure, a large-bore drainage tube was placed intraperitoneally for induction of fecal peritonitis later^5^.

As previously reported^5^, all pressures were measured and displayed on a multi-modular monitor (S/5 Critical Care Monitor; Datex-Ohmeda, GE Healthcare, Helsinki, Finland). Continuous cardiac output (L/min, by thermodilution) was measured with a Vigilance monitor (Baxter Healthcare Corporation, Edwards Critical Care Division, Irvine, CA), and O_2_ saturations were measured continuously with a Vigilance monitor or intermittently with a blood gas analyzer (Radiometer, Copenhagen, Denmark). Blood flows were measured with ultrasonic transit time perivascular flowmeters using double-channel TS 140 420 flowmeters (Transonic Systems Inc., Ithaca, New York, USA). The signals of all the pressures and blood flows were recorded at 100 Hz by a data acquisition system (LabVIEW; National Instruments, Austin, USA).

### Study protocol

After surgical preparation, one-hour stabilization was allowed, followed by baseline blood and cerebrospinal fluid (CSF) sampling and data acquisition within 30 min (time point T1). Then, the pigs were randomized to fecal peritonitis (n = 10)/sham (n = 10) using sealed envelopes and to groups with/without abdominal vacuum, for a separate study.

Fecal peritonitis was induced by peritoneal instillation of 2 g/kg of autologous feces dissolved in 250 mL warmed glucose 5% solution. After 8 h observation, before resuscitation another set of blood and CSF samples were collected (T2), then protocol-based resuscitation was started and continued for three days (resuscitation period, RP). Adequate resuscitation is important, and we used a strict protocol adhering to the current recommendations. Details about the resuscitation protocol can be found in the Supplemental Material.

Further blood and CSF samplings were done just before RP + 24 h (T3), RP + 48 h (T4) and a couple of hours after RP + 72 h (T5). At the end of the experiment, anesthesia was deepened, and the animals were euthanized with 40 mmol potassium chloride.

The protocol is illustrated in Fig. 1.

### Laboratory analyses

At each time point, arterial blood samples were collected, immediately refrigerated-centrifuged and EDTA-plasma was isolated for metabolomics and laboratory analyses. Then we measured hemoglobin concentration, hematocrit, lactate from blood sampling at different sites, concentrations of ammonia, alanine amino transferase (ALAT), aspartate amino transferase (ASAT), platelet count, prothrombin time, leukocytes, band neutrophils, creatinine, creatinine kinase and bilirubin on top of arterial and mixed-venous blood gasses. At each time point, CSF samples were collected for metabolomics and laboratory analyses (e.g. glucose, lactate concentration). We didn’t measure Adenosine triphosphate (ATP) or its products adenosine diphosphate (ADP) and adenosine monophosphate (AMP), as they are related to mitochondrion energy metabolism, they provide a rather global picture, as they pertain to all possible organ cells and they are not so specific for the pathways we considered in our animal model.

### Metabolomics analyses of plasma and cerebrospinal fluid samples

A targeted quantitative approach using combined direct flow injection and liquid chromatography (LC) tandem mass spectrometry (MS/MS) (AbsoluteIDQ 180 kit, Biocrates, Innsbruck, Austria) was employed for the metabolomics analysis of plasma and CSF. The assay quantifies 186 metabolites from six analyte groups: acylcarnitines, amino acids, biogenic amines, hexoses (sum of hexoses), glycerophospholipids and sphingomyelins. The method combines derivatization and extraction of analytes with selective mass-spectrometric detection using multiple reaction monitoring (MRM) pairs. Methodological details and data pre-processing have been reported in our previous articles^10,12^. Measurable metabolites and their extended names and abbreviations are listed in Supplementary Table S1. The concentrations (µM) of all the metabolites measured in the animal experiment (plasma and CSF) are reported in the Supplemental Table S2.

### Multilevel and multivariate analysis of plasma metabolomic data

MultiLevel Simultaneous Component Analysis (MLSCA) and multilevel partial least square discriminant analysis (MLPLSDA) models were proposed by Westerhuis et al.^32^ and De Noord and Theobald^33^. In the multilevel models, the variation between and within subjects are separated. The aim of these analyses is to separate the contribution of subject variability by taking into account the fact that the measures are repeated, i.e. from the same subject. The between-subject variation in multilevel data analysis uses the average of the observations, while the within-subject variation uses the net differences between paired observations. The initial step in the multilevel model is therefore to separate the between-subject from the within-subject variation. We report the key steps of these methods in the supplemental material.

In this study, we applied a MLSCA model on *I* = 10 septic shock animals at the five time points (*K*_*i*_ = 5) using all metabolites concentrations as variables (J = 124), in order to have a global picture about the changes induced by the sepsis over the time of the experiment.

Then we considered the fold-change from T1 to T2 and from T1 to T5, i.e. from baseline to development of sepsis and from baseline to the end of the experiment, in order to characterize the main changes induced by sepsis in septic and sham animals. As the number of features (124 metabolite concentrations) is much higher than the number of observations (20 pigs) we filtered the variable so as to avoid redundancies and collinearities, and considered the binary classification as septic and sham.

We adopted the method proposed by Peng et al., which is minimal-redundancy-maximal-relevance (mRMR)^34^. This algorithm sorts the features according to their relevance to the classification (maximum relevance criterion) and their redundancy (minimum redundancy criterion) in relation to the other variables. The ranking is based on the mutual information between the outcome and each feature and on the mutual information between each couple of features. The features distribution is discretized according to the interquartile range in order to apply the mRMR algorithm and the first 40 ranked features were selected for further analyses. The first 40 ranked features were used in a partial least square discriminant analysis (PLS-DA) model to separate sham and septic animals, and we ranked the features according to the variable importance in projection (VIP) scores, which represent the weights of each feature in the PLS-DA model. A feature with a VIP score higher than 1 is usually considered significant.

### Statistical analysis

The values of each variable are compared for the septic and sham groups at each time point using the Wilcoxon rank-sum test. To deal with the large number of comparisons for the metabolites, we also computed the false discovery rate (FDR). For significant comparisons p < 0.05 and FDR < 0.05 were considered.

As regard metabolomics data, we focused our univariate analysis on specific pathways characterizing the acute phase of shock and the phases of resuscitation, and clearly emerged from the multivariate analyses. For each specific pathway we analyzed also specific ratios commonly adopted in literature to characterize the bioavailability of pathway substrates.

## Supplementary Information

Supplementary Information 1.

Supplementary Information 2.
