# Supplementary material for: Persistent hyperammonia and altered concentrations of urea cycle metabolites in a 5-day swine experiment of sepsis
Source: Sci Rep. 2021 Sep 16;11:18430. doi: 10.1038/s41598-021-97855-7 (PMC8445921; doi:10.1038/s41598-021-97855-7)
Supplement: Supplementary file 1 — Supplementary Information 1. [file 41598_2021_97855_MOESM1_ESM.docx]

**Supplemental material**

**Persistent hyperammonia and altered concentrations of urea cycle metabolites in a 5-day swine experiment of sepsis**

Manuela Ferrario^1*#^, Roberta Pastorelli^2*^, Laura Brunelli^2^, Shengchen Liu^3^, Pedro Paulo Zanella do Amaral Campos^4^, Daniela Casoni^5^, Werner J. Z’Graggen^6^, Stephan M. Jakob^3^

^1^Politecnico di Milano, Department of Electronics, Information and Bioengineering, Milano, Italy

^2^Istituto di Ricerche Farmacologiche Mario Negri IRCCS, Milano, Italy

^3^Department of Intensive Care Medicine, Bern University Hospital, University of Bern, Bern, Switzerland

^4^Department of Intensive Care Unit, Hospital Israelita Albert Einstein, São Paulo, Brazil

^5^Experimental Surgery Facility, Department for Bio Medical Research, University of Bern, Bern, Switzerland

^6^Departments of Neurology and Neurosurgery, Bern University Hospital, University of Bern, Bern, Switzerland

* contributed equally to the work

^#^corresponding author: manuela.ferrario@polimi.it

**Table S1**. List of the measurable metabolites using the Biocrates Absolute IDQ p180 platform.

Aa, acyl-acyl; ae, acyl-alkyl; a, lyso; Cx:y, where x is the number of carbons in the fatty acid side chain; y is the number of double bonds in the fatty acid side chain; DC, decarboxyl; M methyl; OH, hydroxyl; PC, phosphatidylcholine; SM, sphingomyeline

| **mETABOLITE CLASS** | **#** | **mETABOLITE NAME OR ABBREVIATION** | **Biological relevance**  **(SELECTED EXAMPLES)** |
| --- | --- | --- | --- |
| **Amino acids** | 21 | Alanine, arginine, aspartate, citrulline, glutamine, glutamate, glycine, histidine, isoleucine, leucine, lysine, methionine, ornithine, phenylalanine, proline, serine, threonine, tryptophan, tyrosine, valine | Amino acid metabolism, urea cycle, activity of gluconeogenesis and glycolysis, insulin sensitivity, neurotransmitter metabolism, oxidative stress |
| **Carnitine** | 1 | C0 | Energy metabolism, fatty acid transport and mitochondrial fatty acid oxidation, ketosis, oxidative stress, mitochondrial membrane damage |
| **Acylcarnitine** | 39 | C2, C3, C3:1, C3-OH, C4, C4:1, C4-OH, C5, C5:1, C5:1-DC, C5-DC, C5-M-DC, C5-OH, C6, C6:1, C7-DC, C8, C9, C10, C10:1, C10:2, C12, C12-DC, C14, C14:1, C14:1-OH, C14:2, C14:2-OH, C16, C16:1, C16:1-OH, C16:2, C16:2-OH, C16-OH, C18, C18:1, C18:1-OH, C18:2 |  |
| **Biogenic amines** | 19 | Acetylornithine, asymmetric dimethylarginine, total dimethylarginine, alpha-aminoadipic acid, carnosine, creatinine, histamine, kynurenine, methionine sulfoxide, nitrotyrosine, hydroxyproline, phenylethylamine, putrescine, sarcosine, serotonin, spermidine, spermine, taurine | Neurological disorders, cell proliferation, cell cycle progression, DNA stability, oxidative stress |
| **Lyso-phosphatidylcholines** | 14 | lysoPC a C14:0 / C16:0/ C16:1/ C17:0/ C18:0/ C18:1/ C18:2/ C20:3/ C20:4/ C26:0/ C26:1/ C28:0/ C28:1 | Degradation of phospholipids, membrane damage, signaling cascades, fatty acid profile |
| **Diacyl-phosphatidylcholines** | 38 | PC aa C24:0/ C26:0/ C28:1/ C30:0/ C30:2/ C32:0/ C32:1/ C32:2/ C32:3/ C34:1/ C32:2/ C34:3/ C32:4/ C36:0/ C36:1/ C36:2/ C36:3/ C36:4/ C36:5/ C36:6/ C38:0/ C38:1/ C38:3/ C38:4/ C38:5/ C38:6/ C40:1/ C40:2/ C40:3/ C40:4/ C40:5/ C40:6/ C42:0/ C42:1/ C42:2/ C42:4/ C42:5/ C42:6 | Dyslipidemia, membrane composition and damage, fatty acid profile, activity of desaturases |
| **Acyl-alkyl-phosphatidylcholine** | 38 | PC ae C30:0/ C30:2/ C32:1/ C32:2/ C34:0/ C34:1/ C34:2/ C34:3/ C36:0/ C36:1/ C36:2/ C36:3/C36:4/C36:5/C38:0/C38:1/C38:2/C38:3/C38:4/C38:5/C38:6/C40:1/C40:2/C40:3/ C40:4/C40:5/C40:6/C42:0/C42:1/C42:2/C42:3/C42:4/C42:5/C44:3/C44:4/C44:5/C44:6 |  |
| **Sphingomyelins** | 15 | SM (OH) C14:1, SM C16:0, SM C16:1, SM C16:1, SM C18:0, SM C18:1, SM C20:2, SM C22:3, SM (OH) C22:1, SM (OH) C22:2, SM C24:0, SM C24:1, SM (OH) C24:1, SM C26:0, SM C26:1 | Signaling cascades, membrane damage (eg. neurodegeneration) |
| **Hexose** | 1 | H1 | Carbohydrate metabolism |
| **Total** | 186 |  |  |

List of abbreviations used: Ala, Alanine; Arg, Arginine; Asn, Asparagine; Asp, Aspartate; Cit, Citrulline; Gln, Glutamine; Glu, Glutamate; Gly, Glycine; His, Histidine; Ile, Isoleucine; Leu, Leucine; Lys, Lysine; Met, Methionine; Orn, Ornithine; Phe, Phenylalanine; Pro, Proline; Ser, Serine; Thr, Threonine; Trp, Tryptophan; Tyr, Tyrosine; Val, Valine; Ac-Orn, Acetylornithine; total DMA, total dimethylarginine; ADMA, Asymmetric dimethylarginine; SDMA, Symmetric dimethylarginine; alpha-AAA, alpha-Aminoadipic acid; Met-SO, Methioninesulfoxide; Nitro-Tyr, Nitrotyrosine; c4-OH-Pro, cis-4-Hydroxyproline; t4-OH-Pro, trans-4Hydroxyproline; PEA , Phenylethylamine

**Table S2 -** Concentrations (µM) of all the metabolites measured in the animal experiment (plasma and CSF)

**excel file**

**Figure S1-** Boxplot of the hemodynamic measures at each time for the two groups. The hemodynamic indices are heart rate (HR), mean arterial pressure (MAP), central venous pressure (CVP), pulmonary artery pressure (PAP), core temperature measured in pulmonary artery, cardiac output (CO) calculated by thermodilution, pulmonary wedge pressure (PWP), carotid O_2_ saturation, mixed venous oxygen saturation (SvO_2_).

# p <0.05, ^o^ p < 0.01 sham vs sepsis(Wilcoxon rank-sum test).

**Figure S2-** Boxplot of the concentrations of coagulation and inflammation markers. WBC: white blood cell counts, # p <0.05, ° p < 0.01 sham vs sepsis (Wilcoxon rank-sum test).

**Table S3-** Medians (25-75th) values of the hemodynamic measures and laboratory analyses at each time for the two groups. The hemodynamic indices are heart rate (HR), mean arterial pressure (MAP), central venous pressure (CVP), pulmonary artery pressure (PAP), core temperature in pulmonary artery, cardiac output (CO) calculated by thermodilution, pulmonary wedge pressure (PWP), carotid O2 saturation, mixed venous oxygen saturation (SvO2), Hematocrit (Htc), Hemoglobin (Hb), platelets, white blood cells (WBC), prothrombin time (PT), bilirubin, ammonia, alanine amino transferase (ALAT), aspartate amino transferase (ASAT), creatine kinase (CK), band neutrophils percentage, lactate levels measured at different sites (carotid, pulmonary artery PA, portal vein PV, hepatic artery HA, renal vein RV, superior sagittal sinus SSS).

# p <0.05, °p <0.01 sham SH vs sepsis SS (Wilcoxon rank-sum test).

|  |  | **T1** | **T2** | **T3** | **T4** | **T5** |
| --- | --- | --- | --- | --- | --- | --- |
| **HR(bpm)** | SS | 99.2 (90.0, 108.0) | 183.0 (170.4, 200.8)° | 166.5 (123.0, 178.0)° | 141.0 (105.0, 148.0)° | 119.5 (95.9, 133.0)° |
|  | SH | 98.3 (92.0, 104.0) | 83.5 (80.0, 108.0) | 72.0 (67.0, 107.0) | 67.0 (53.0, 82.4) | 68.5 (62.0, 72.0) |
| **MAP (mmHg)** | SS | 79.3 (70.2, 84.0) | 62.6 (48.0, 72.0) | 70.6 (66.7, 75.0) | 71.4 (69.0, 74.1) | 72.3 (70.0, 76.0) |
|  | SH | 75.1 (71.0, 80.0) | 71.8 (68.1, 77.0) | 71.0 (68.3, 82.0) | 76.1 (67.5, 79.0) | 71.3 (69.0, 80.0) |
| **CVP (mmHg)** | SS | 3.9 (1.8, 6.6) | 4.2 (1.7, 6.0) | 5.6 (3.4, 8.2) | 7.4 (6.5, 9.8) | 9.0 (7.0, 11.0) |
|  | SH | 5.5 (3.0, 6.7) | 5.5 (4.7, 7.0) | 8.0 (6.0, 8.0) | 7.4 (7.0, 8.9) | 7.0 (6.0, 8.0) |
| **PAP (mmHg)** | SS | 18.4 (14.4, 20.0) | 21.5 (16.3, 23.0) | 23.5 (18.2, 25.3) | 23.9 (20.2, 28.0)# | 26.5 (21.1, 29.6) |
|  | SH | 18.7 (18.0, 20.0) | 17.0 (17.0, 18.3) | 19.8 (18.0, 22.0) | 19.5 (18.0, 22.0) | 21.0 (21.0, 22.3) |
| **Temp (°C)** | SS | 40.1 (39.6, 40.7) | 41.4 (41.1, 42.0)° | 40.3 (39.7, 40.7)° | 39.6 (39.2, 40.3)° | 39.8 (39.4, 40.7)° |
|  | SH | 40.0 (38.9, 40.5) | 39.6 (39.4, 39.7) | 38.7 (38.3, 39.1) | 37.9 (37.5, 38.4) | 38.3 (38.0, 38.5) |
| **CO (L/min)** | SS | 4.3 (3.9, 5.3) | 3.1 (2.5, 4.0)# | 6.1 (5.0, 7.3) | 6.5 (6.0, 7.8)# | 6.5 (5.9, 7.9)° |
|  | SH | 4.5 (3.9, 5.3) | 3.9 (3.6, 4.8) | 4.6 (4.1, 6.0) | 5.1 (3.6, 5.3) | 4.4 (4.2, 5.1) |
| **PWP (mmHg)** | SS | 5.5 (2.0, 7.0) | 4.0 (2.8, 6.0)° | 6.0 (3.0, 9.0) | 9.0 (8.0, 11.0) | 9.5 (9.0, 12.0) |
|  | SH | 5.0 (4.0, 6.0) | 7.0 (6.0, 8.0) | 8.0 (7.0, 10.0) | 8.0 (8.0, 9.0) | 8.0 (8.0, 10.0) |
| **SaO2 (%)** | SS | 97.2 (96.8, 98.0) | 95.2 (94.6, 95.7)# | 94.8 (93.8, 97.0)° | 98.5 (95.0, 100.0) | 99.2 (93.0, 100.0) |
|  | SH | 97.3 (94.5, 98.5) | 97.4 (96.8, 98.5) | 98.4 (97.9, 100.0) | 100.0 (99.1, 100.0) | 100.0 (98.4, 100.0) |
| **SvO2 (%)** | SS | 50.2 (46.5, 55.0) | 48.0 (43.4, 57.0) | 64.7 (62.9, 68.0) | 66.8 (63.0, 67.6)# | 60.2 (57.6, 65.0) |
|  | SH | 47.9 (44.1, 52.3) | 51.2 (49.0, 53.1) | 56.5 (55.8, 67.9) | 56.0 (52.3, 58.0) | 57.1 (53.1, 62.0) |
| **Htc (L/L)** | SS | 0.30 (0.28, 0.31) | 0.39 (0.36, 0.42)° | 0.29 (0.28, 0.31)° | 0.24 (0.23, 0.25)# | 0.21 (0.19, 0.22) |
|  | SH | 0.29 (0.28, 0.32) | 0.27 (0.25, 0.29) | 0.23 (0.22, 0.23) | 0.20 (0.17, 0.23) | 0.21 (0.19, 0.26) |
| **Hb (g/L)** | SS | 101 (91, 102) | 134 (120, 142)° | 98 (93, 105)° | 84 (76, 86)# | 72 (62, 76) |
|  | SH | 98.5 (92.0, 105.0) | 88.5 (83.0, 95.0) | 77.0 (72.0, 79.0) | 65.0 (57.0, 77.0) | 68.0 (63.0, 84.0) |
| **Platelets (10^9/L)** | SS | 299 (247, 474) | 193 (176, 224) | 86 (60, 94)° | 106 (69, 118)° | 148 (112, 183)° |
|  | SH | 295 (222, 393) | 227 (171, 337) | 178 (148, 269) | 193 (156, 226) | 234 (217, 262) |
| **WBC (10^9/L)** | SS | 19.3 (16.7, 23.8) | 11.8 (11.2, 13.8)# | 13.8 (11.1, 19.1) | 12.2 (10.1, 13.7) | 10.3 (8.6, 12.7) |
|  | SH | 20.9 (18.4, 27.1) | 18.4 (12.7, 19.6) | 14.0 (10.5, 15.7) | 12.2 (9.1, 14.1) | 11.8 (9.6, 15.9) |
| **Creatinine (µmol/L)** | SS | 95.5 (87.0, 98.0) | 166.5 (141.0, 182.0)° | 112.5 (105.0, 165.0)° | 94.5 (82.0, 110.0)# | 87.5 (83.0, 92.0) |
|  | SH | 90.5 (74.0, 100.0) | 107.5 (99.0, 110.0) | 86.5 (79.0, 97.0) | 79.5 (65.0, 96.0) | 77.0 (71.0, 86.0) |
| **PT (sec)** | SS | 13.9 (13.3, 14.3) | 14.7 (14.4, 16.2)# | 14.6 (14.0, 15.0) | 14.0 (13.5, 14.5) | 14.1 (13.8, 14.7)# |
|  | SH | 13.8 (12.8, 14.0) | 14.0 (13.6, 14.4) | 14.2 (13.4, 14.5) | 13.5 (13.2, 13.6) | 13.6 (13.0, 13.7) |
| **Bilirubin (µmol/L)** | SS | 1.2 (0.8, 1.6) | 1.4 (1.2, 2.4) | 1.4 (1.3, 1.6) | 1.2 (0.8, 1.3) | 1.2 (0.9, 2.1)# |
|  | SH | 1.2 (0.6, 1.9) | 1.1 (0.7, 1.5) | 1.1 (0.6, 1.9) | 0.6 (0.3, 0.9) | 0.7 (0.4, 1.1) |
| **Ammonia (µmol/L)** | SS | 50.5 (38.0, 64.0) | 107.0 (83.0, 124.5)° | 86.0 (70.5, 94.2)° | 76.5 (62.0, 86.0)° | 102.5 (89.0, 114.0)° |
|  | SH | 50.0 (47.5, 53.0) | 31.5 (21.0, 36.0) | 38.0 (31.8, 46.2) | 44.5 (40.0, 55.0) | 53.5 (49.0, 70.0) |
| **ALAT (IU/L)** | SS | 30.5 (27.0, 46.0) | 27.0 (25.0, 35.0) | 31.0 (29.0, 46.0)° | 28.5 (24.0, 47.0)° | 28.0 (21.0, 34.0) |
|  | SH | 33.0 (27.0, 34.0) | 28.0 (25.0, 29.0) | 23.0 (20.0, 24.0) | 21.5 (18.0, 24.0) | 22.0 (20.0, 25.0) |
| **ASAT (IU/L)** | SS | 53.0 (41.0, 67.0) | 67.0 (46.0, 137.0)# | 115.0 (82.0, 138.0)° | 80.0 (60.0, 92.0)° | 55.0 (45.0, 65.0)° |
|  | SH | 69.0 (28.0, 88.0) | 41.0 (23.0, 51.0) | 32.5 (19.0, 37.0) | 26.5 (19.0, 30.0) | 22.5 (18.0, 39.0) |
| **CK (IU/L)** | SS | 854 (691, 1064) | 806 (715, 1109) | 1144 (784, 1372) | 584 (461, 722)# | 392 (306, 438)° |
|  | SH | 1249 (646, 1750) | 874 (716, 1022) | 886 (713, 1178) | 834 (690, 1181) | 836 (646, 1595) |
| **Band neutrophils (%)** | SS | 2.5 (0.4, 12.0) | 50.5 (40.0, 59.0)° | 33.2 (27.0, 47.0)° | 14.0 (11.5, 18.5)° | 6.0 (1.5, 8.5)° |
|  | SH | 6.8 (4.0, 15.0) | 0.2 (0.0, 4.5) | 0.8 (0.0, 3.0) | 0.5 (0.0, 1.0) | 0.0 (0.0, 0.5) |
| **Lactate (mmol/L)** | SS | 0.60 (0.50, 0.70) | 0.80 (0.70, 1.20)° | 0.80 (0.60, 1.00)° | 0.65 (0.50, 0.80)° | 0.45 (0.40, 0.70)° |
|  | SH | 0.60 (0.50, 0.80) | 0.40 (0.40, 0.50) | 0.35 (0.30, 0.50) | 0.30 (0.20, 0.30) | 0.30 (0.20, 0.30) |
| **Lactate PA (mmol/L)** | SS | 0.60 (0.50, 0.70) | 0.85 (0.70, 1.30)° | 0.70 (0.60, 1.10)° | 0.60 (0.50, 0.80)° | 0.45 (0.40, 0.80)° |
|  | SH | 0.60 (0.50, 0.80) | 0.40 (0.40, 0.60) | 0.35 (0.30, 0.50) | 0.30 (0.20, 0.30) | 0.30 (0.20, 0.40) |
| **Lactate PV (mmol/L)** | SS | 0.70 (0.60, 0.80) | 1.25 (1.10, 1.40)° | 1.00 (0.80, 1.30)° | 0.90 (0.70, 1.05)° | 0.75 (0.50, 1.00)° |
|  | SH | 0.75 (0.70, 1.00) | 0.55 (0.50, 0.70) | 0.45 (0.40, 0.60) | 0.40 (0.30, 0.50) | 0.40 (0.38, 0.40) |
| **Lactate HV (mmol/L)** | SS | 0.30 (0.20, 0.43) | 0.40 (0.30, 0.55) | 0.40 (0.25, 0.50)# | 0.40 (0.30, 0.62)° | 0.30 (0.20, 0.57) |
|  | SH | 0.30 (0.18, 0.55) | 0.30 (0.15, 0.40) | 0.10 (0.00, 0.30) | 0.10 (0.00, 0.20) | 0.20 (0.10, 0.30) |
| **Lactate RV(mmol/L)** | SS | 0.50 (0.40, 0.50) | 0.70 (0.47, 0.95)° | 0.60 (0.50, 0.70)# | 0.60 (0.50, 0.60)° | 0.40 (0.30, 0.65)° |
|  | SH | 0.50 (0.40, 0.70) | 0.35 (0.20, 0.50) | 0.30 (0.20, 0.40) | 0.30 (0.20, 0.35) | 0.25 (0.20, 0.30) |
| **Lactate SSS (mmol/L)** | SS | 0.65 (0.55, 0.70) | 0.85 (0.75, 1.00)° | 0.75 (0.70, 1.10)° | 0.60 (0.60, 0.80)# | 0.50 (0.47, 0.70)° |
|  | SH | 0.70 (0.60, 0.88) | 0.40 (0.38, 0.62) | 0.45 (0.40, 0.60) | 0.35 (0.30, 0.40) | 0.35 (0.30, 0.45) |

**Figure S3 -** Boxplot of the plasma concentrations of some lysoPCs, total PCs and the sum of the most abundant lysoPCs (lysoPC 16:X and lysoPC 18:X) at each time for the two groups: sham and septic pigs. # p <0.05, ^o^p- <0.01 (Wilcoxon rank-sum test) and FDR<0.05 sham vs sepsis; µM=µmol/L. The lysoPC and PC species decreased after the insult and stayed significantly lower in the septic group than in sham animals.

**Figure S4 -** Boxplot of the plasma concentrations of sugars (hexoses), alanine, glucogenic amino acids (AA) and lactate in different circulatory districts at each time for sham and septic pigs. # p <0.05 (Wilcoxon rank-sum test) and FDR<0.05 sham vs sepsis; µM=µmol/L. All septic animals had increased lactate levels ,with a first increase of alanine and glucogenic AA at T2.

**Resuscitation protocol**

After peritonitis induction, all animals were submitted to an observation period of 8 hours without resuscitation. At the end of observation period, a resuscitation period of approximately 76 hours were performed, including fluid infusion, circulation support, electrolyte maintenance, antibiotic therapy and reduction of body temperature, etc.

During the resuscitation period the sum of ringer lactate solution (RL), G50% solution and enteral nutrition infusion rates were adjusted to keep the total infusion rate of 3.0 ml/kg/h.

Additional fluid boluses were given to treat hypovolemia. In particular, after 150ml bolus of RL was given, if cardiac output or stroke volume increased more than 10%, the fluid response was considered positive, otherwise repeated fluid bolus were given until a positive response

Noradrenaline was given to increase systemic vascular resistance and to keep mean arterial pressure higher than 65mmHg.

The blood glucose was maintained between 3.5-7.0mmol/L through GS 50% or insulin infusion.

The PH was maintained between 7.35-7.45 via adjusting the respiratory rate and minute ventilation. The arterial oxygen partial pressure was maintained between 100-150mmHg and oxygen saturation >90% by tracheal suctioning, recruitment maneuver or by setting new PEEP/FiO2.

Zinacef 1.5 g was infused during anesthesia induction as prophylaxis for surgery. Piperacillin-tazobactam 2.25g was administered intravenously (IV) in an 8 hours interval.

Liquemin 10000IU/24h was infused IV to prevent deep vein thrombosis.

The animal was cooled when core temperature was higher than 39.5°C by fan, air conditioner, alcohol spray or ice bags.

**Multivariate and multilevel analyses of metabolomics data**

Here we recall the key steps for the computation of the MultiLevel Simultaneous Component Analysis (MLSCA) and the MultiLevel Partial Least Square Discriminant Analysis (MLPLSDA) model.

The total data matrix ***X*** (*N*×*J*) contains measurements for *I* subjects observed at *K_i_* time points on J variables, where the total number of observations is $N=\sum_{i=1}^{I} K_{i}$. An element $x_{ijk_{i}}$of matrix X, which contains a measurement of subject *i* on variable *j* at time point k_i_, can be decomposed as

$x_{ijk_{i}}=x_{.j.}+\left( x_{ij.}-x_{.j.} \right)+(x_{ijk_{i}}-x_{ij.})$ (1)

where $x_{.j.}$ is the overall mean for variable *j* and $x_{ij.}$ is the mean of subject *i* on variable *j*. The first term in Equation (1) is an offset that is constant across subjects and time points, the second term is the between-subject deviation, and the third term describes the within-subject deviation. Similarly, the sum of squares per variable can be separated in three parts, analogous to Analysis of Variance. The objective of this two-level model is to approximate the data, explaining the offset, between-subject, and within-subject variation as well as possible.

In the multilevel MLSCA model we assumed that the within –loadings matrices are the same for all the subjects. The MLSCA model for a subject *i* with data matrix ***X****_i_* (*K_i_* ×*J*) containing the part of **X** with data from subject *i* (where *i*=1,…, *I*) is the following:

$\boldsymbol{X}_{i}=\boldsymbol{1}_{K_{i}}\boldsymbol{m}^{'}+\boldsymbol{1}_{K_{i}}{\boldsymbol{t}'}_{b,i}{\boldsymbol{P}'}_{b}+\boldsymbol{T}_{w,i}{\boldsymbol{P}'}_{w}+\boldsymbol{E}_{i}$ (2)

where $\boldsymbol{1}_{K_{i}}$ is a *K_i_* ×1 vector of ones, **m** (*J*×1) contains the offsets of the J variables, $\boldsymbol{t}_{b,i}$ (*R_b_*×1) contains the between-subject scores of subject *i* for *R_b_* retained between-components, $\boldsymbol{P}_{b}$ (*J*×*R_b_*) is the between-subject loading matrix, $\boldsymbol{T}_{w,i}$ (*K_i_*×*R_w_*) contains the within-subject scores for subject *i* for *R_w_* retained within-components, $\boldsymbol{P}_{w}$ (*J*×*R_w_*) is the within-subject loadings matrix and $\boldsymbol{E}_{i}$ (*K_i_*×*J*) contains the residual.

In the multilevel MLSCA the offset, the between-subject loadings, and the within-subject loadings are assumed equal for all subjects, and the within-subject loadings are assumed to be time invariant as well. This means that interpretation of the components is equal for all subjects. The between-subject and within-subject variations in the data are described in separate terms, and are thus not confounded as in ordinary PCA. Given these hypotheses, the three parts of the model can be solved separately.

The between-subject part can be obtained from the matrices *X_b_* (*I* ×*J*), which contain the mean vectors of the *I* subjects, by means of the singular value decomposition (SVD). The within-subject part of the model can be obtained from the matrix *X_w_* (*N*×*J*), which consists of the concatenated centered matrices ***X****i*, by means of the singular value decomposition. In the multilevel partial least square discriminant analysis (PLS-DA), instead of using the simple SVD as in MLSCA, the projections are guided by the outcomes (**Y** matrix).

**Figure S5 -** Boxplot of ammonia blood concentration, urea cycle-related metabolites and their ratios from plasma samples at each time point for sham and septic pigs. # p <0.05, ^o^ p <0.01 (Wilcoxon rank-sum test) sham vs shock; µM=µmol/L.
